# Trends in Public Interest in Digital Musculoskeletal Care: A Google Trends Analysis of Hinge Health From 2020 to 2025

**DOI:** 10.7759/cureus.91136

**Published:** 2025-08-27

**Authors:** Nikhil Maddina, Albert D Mousad, Fawaz Ibikunle, Minna Hassaballa, Shantanu Amin, Nicole Yeroushalmi

**Affiliations:** 1 College of Osteopathic Medicine, Midwestern University Chicago College of Osteopathic Medicine, Downers Grove, USA; 2 Orthopedics, Geisinger Musculoskeletal Institute, Wilkes-Barre, USA; 3 College of Osteopathic Medicine, Kansas City University College of Osteopathic Medicine, Joplin, USA; 4 Medical School, City University of New York (CUNY) School of Medicine, New York, USA

**Keywords:** chronic pain, healthcare access disparity, hinge health, infodemiology, musculoskeletal disorders, telemedicine

## Abstract

Introduction

Hinge Health (Hinge Health, Inc., San Francisco, CA) and similar musculoskeletal (MSK) telehealth platforms have grown rapidly in recent years, offering a new avenue for accessible, at-home care. As digital health further integrates into routine clinical practice, understanding public interest in these services can provide insight into shifting care-seeking behaviors and potential disparities in healthcare access. This study aims to analyze Google Trends (Google LLC, Mountain View, CA) data to evaluate temporal and geographic patterns of public search interest in Hinge Health in the United States, comparing upper and lower extremity musculoskeletal injuries, with the hypothesis that interest has increased over time.

Methods

Google Trends was used to extract relative search volume (RSV) data for the term “Hinge Health” and related MSK keywords (e.g., “chronic pain,” “knee pain”) between January 2020 and April 2025. RSV data was analyzed between two intervals (January 2020-August 2022) and (September 2022-April 2025) using independent t-tests to evaluate changes over time. Geographic patterns were analyzed and visualized using heat maps to identify regions with elevated interest.

Results

Search trends for “Hinge Health” increased from January 2020 to April 2025. The median RSV rose from 23.5 in the first interval to 54.5 in the second, indicating a statistically significant difference (P < 0.001). States with the highest RSV included Minnesota, California, Oregon, and Colorado, while the lowest were Mississippi, Nebraska, Louisiana, and West Virginia. Public interest in upper and lower body MSK pain also increased across most terms. Conditions like chronic neck (P = 0.043), shoulder (P < 0.001), back (P < 0.001), knee (P < 0.001), and pelvic pain (P < 0.001) showed significant growth in RSV between intervals. Chronic shin pain was the only category that did not change significantly (P = 0.055). Geographic differences were also notable. States such as Kentucky, Connecticut, and Arkansas showed the greatest increases in RSV over time, while states like Montana, Hawaii, and the District of Columbia saw declines.

Conclusion

Digital public interest in Hinge Health has increased significantly from 2020 to 2025, reflecting broader changes in healthcare delivery and access during the post-COVID period. However, the geographic and socioeconomic disparities in engagement reveal ongoing inequalities in digital healthcare access. Future efforts to expand digital access to MSK care may focus on building socioeconomic infrastructure, improving digital literacy in rural regions, community education, and establishing clearer policy frameworks to promote equitable integration of telehealth services nationwide.

## Introduction

Chronic musculoskeletal (MSK) conditions are among the leading causes of disability in the United States, yet access to timely, in-person care remains limited [1]. Patients in traditional healthcare models face long wait times for specialist visits, imaging follow-ups, and physical therapy, which often delay treatment and exacerbate symptoms [2]. These challenges are more difficult in rural areas and among patients with financial or mobility constraints. As a result, more individuals are turning to telehealth platforms for convenience and lower-cost alternatives. 

In response to these challenges, digital MSK health platforms like Hinge Health (Hinge Health, Inc., San Francisco, CA) have gained traction. Hinge Health, founded in 2014 in the United States, offers both virtual physical therapy and wearable pain relief devices, among other remote health technologies. The platform has demonstrated clinical effectiveness through randomized control trials, which reported nearly 68% reduction in pain from the patient’s baseline, and similar studies found nearly 50% lower surgical intervention rates while reducing opioid prescription initiation by 42% [3-5]. Moreover, Hinge Health's initial public offering in March 2025 at a valuation of $2.6 billion indicates public and commercial interest for digital-first approaches in MSK chronic care management [6].

Prior studies support the clinical and behavioral benefits of digital MSK telehealth platforms for MSK conditions. Notably, adherence to exercise regimens has been shown to be higher with telehealth-based rehabilitation compared to non-digital rehabilitation options [7]. These improvements in engagement do not appear to come at the expense of quality; patient outcomes from online MSK rehabilitation programs are non-inferior to those from in-person programs [8]. Furthermore, telehealth utilization remained 38 times higher than pre-pandemic levels by late 2021, demonstrating consistent patient interest in engaging with virtual care models [9]. However, while clinical outcomes and patient engagement with digital MSK platforms are increasingly well documented, less is known about how public interest in these services has evolved over time or varies across regions and condition types. 

This study aims to analyze data from Google Trends (Google LLC, Mountain View, CA) to evaluate temporal and geographic patterns of public search interest in Hinge Health in the United States, comparing upper and lower extremity musculoskeletal injuries, with the hypothesis that interest has increased over time.

## Materials and methods

Google Trends was the primary application used to measure the popularity of search trends over time and across geographic regions. Prior studies have demonstrated its ability to track public interest in orthopedic procedures like knee, hip, and hand surgeries, which often correlate with actual increases in interventions and visits [10,11]. These studies support Google Trends’ use as a valid method to assess public interest and potential clinical impacts in healthcare delivery. Google Trends reports “relative search volume” (RSV), a scaled numerical value that is registered by Google Trends from 0-100. The search application standardizes the popularity of a search term relative to its peak popularity within a specified timeframe. The benefits of this standardization allow for simplified visualization and wide comparison across various terms and locations, even if the search volume varies significantly [12]. 

The search time frame was between January 2020 to April 2025. This was the selected study window because this period helps capture the public engagement from the beginning of the impact of the COVID-19 pandemic, when digital MSK care surged due to telehealth adoption. The settings for the Google Trends search were the specific term in the search bar, followed by the select filters: “United States”, “all categories”, and “web search.” There were two separate data sets collected. The first data set was the RSV of all selected terms over the study’s timeframe to identify any temporal trends. The second data set was the RSV for each search term for all encompassing regions of the United States (to account for 50 states and the capital of Washington, D.C).

The search terms for this study were categorized into two parts. The first part was to search for the MSK company’s name, “Hinge Health” with quotations. The second part was to assess common MSK pathologies that Hinge Health treats. The search terms were categorized into two groups: Upper Body (UB) and Lower Body (LB). The search terms for the UB group are “Hinge Health” + chronic back pain, “Hinge Health” + chronic shoulder pain, and “Hinge Health” + chronic neck pain. The search terms for the LB group are “Hinge Health” + chronic pelvic pain and “ Hinge Health” + chronic knee pain, and “Hinge Health” + chronic shin pain. These regions were included because Hinge Health’s platform explicitly references these body parts when generating personalized care plans and managing multi-region pain [13]. The term “chronic” was included alongside regional MSK body parts to reflect Hinge Health’s focus on long-term conditions, as the company markets itself as a digital solution for delivering significant improvements in chronic pain management [13]. Other body regions were excluded when preliminary results showed insufficient search volume to generate data.

Relative search volume files were extracted from Google Trends for all search terms and analyzed using the median and interquartile range. Additionally, this included the 51 regions of the United States and their respective changes in RSV. The median was reported, given its statistical resistance to outliers, as being less heavily affected by transient RSV spikes. Data was collected across two consecutive 32-month periods: the first period is January 2020 to August 2022, and the second period is September 2022 to April 2025. These timeframes were selected to capture two distinct phases: the initial surge in telehealth and remote MSK care during the COVID-19 pandemic, and the post-pandemic phase of sustained digital platform use. August 2022 was chosen as the split point to evenly divide the dataset for comparative analysis, while April 2025 marked the most recent month with a complete dataset available. Queries were repeated under identical parameters, yielding consistent results. 

Monthly RSV averages were compared using independent t-tests to evaluate changes over time since the two intervals represented non-overlapping independent samples, with an a priori α level set at 0.05 to establish statistical significance. Geographic patterns were also analyzed and visualized using heat maps to identify regions with elevated interest. State-level RSVs for the UB and LB groups were derived by aggregating the RSVs for all relevant subgroups within each category and calculating the median RSV. All statistical analysis was conducted using Microsoft Excel for Microsoft 365 (Microsoft Corporation, Redmond, WA, USA).

## Results

**Figure 1:**
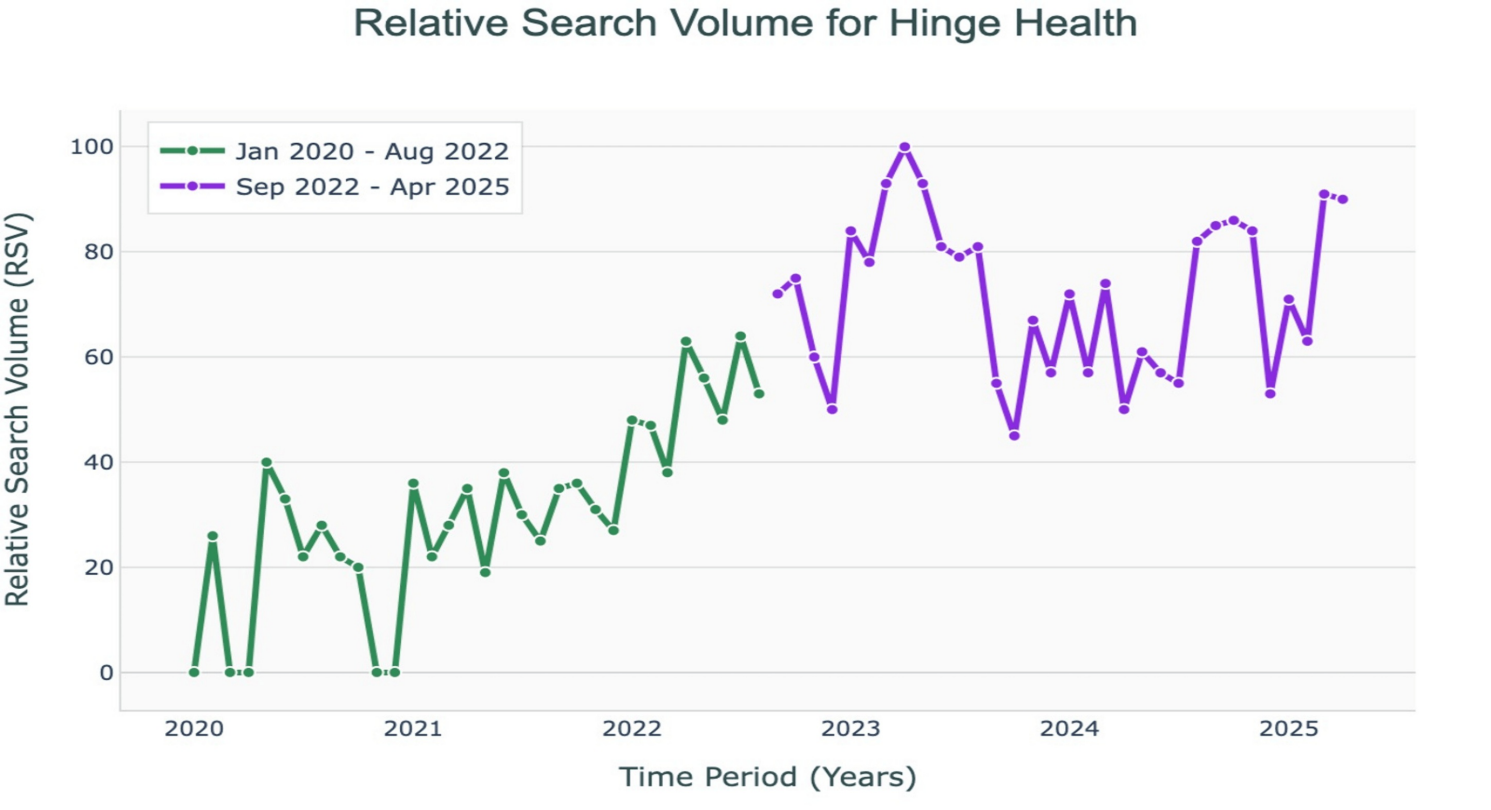
Relative search volume for Hinge Health Relative search volume (RSV) for Hinge Health that was extracted from Google Trends. Shows a trending increase in RSV value since January 2020 to April 2025.

**Figure 2:**
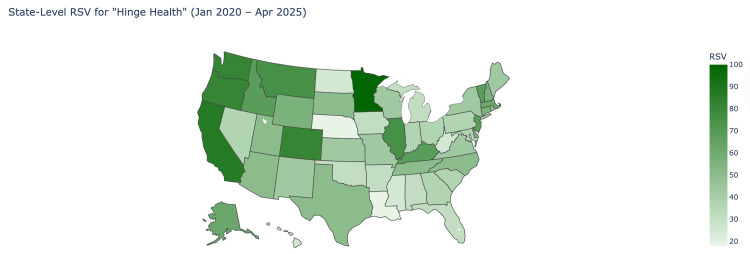
State-level relative search volume for "Hinge Health" (January 2020-April 2025) This is a heat map visualization of the United States where the relative search volume (RSV) of “Hinge Health is shown by how dark the shade of the respective state is, according to the legend.

**Figure 3:**
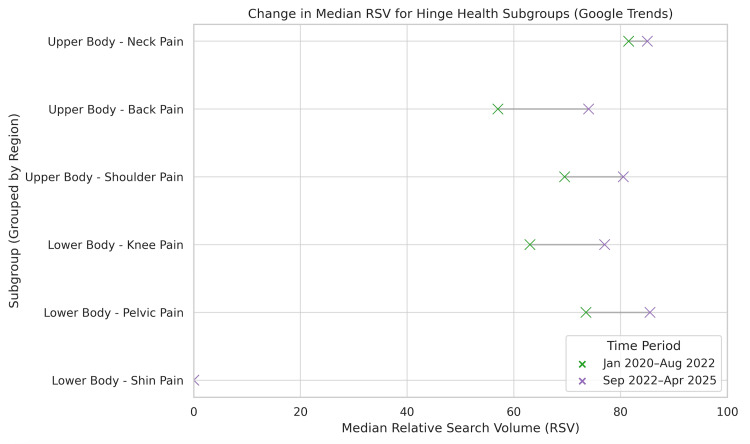
Change in median relative search volume for Hinge Health subgroups This figure shows the median relative search volume per search term across two time periods: January 2020 to August 2022 and September 2022 to April 2025. The green marker shows the first period, and the purple marker shows the second period.

The Google Trends analysis showed that relative search volume for Hinge Health progressed significantly upward from 2020 to 2025 (Figure 1). The median RSV for Hinge Health from January 2020 to April 2025 was 45.5 (IQR: 23.75-54.25). The median RSV for the first interval was 23.5 (IQR: 21.5-30.75), which rose to 54.5 (IQR: 49.75-62.0) in the second interval, which showed a significant difference (P<0.001). The regions that showed the highest RSV were Minnesota (100), California (87), Oregon (81), and Colorado (81) (Figure 3). The regions that showed the least amount of RSV were West Virginia (25), Mississippi (25), Nebraska (18), and Louisiana (18) (Figure 2). 

**Figure 4:**
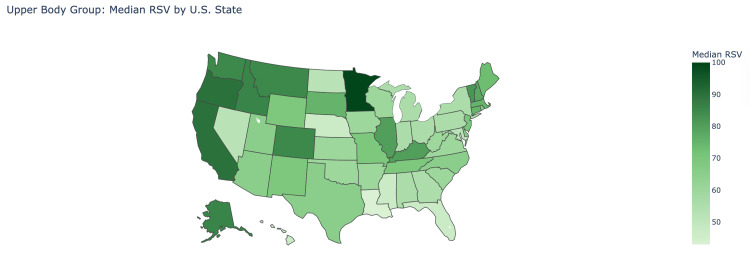
Upper body group: median relative search volume by U.S. state This is a heat map visualization of the United States where the median relative search volume (RSV) of the upper body group is shown by how dark the shade of the respective state is, according to the legend. The darker the shade of green, the higher the median RSV value is in the state.

The median RSV for overall upper body injuries in the first interval was 70 (IQR: 57.75-78.25), which then rose significantly to 80 (IQR: 74-86) in the second interval (P<0.001). There was a similar trend seen throughout each search term in the upper body category. As seen in the chronic neck pain group, the median RSV rose significantly from 81.5 (IQR: 76-86) in the first interval to 85 (IQR: 80-91) in the second interval (P=0.043) (Figure 3). Likewise, the chronic shoulder pain and chronic back pain groups saw significant increases in median RSV from 69.5 (67.75-75.25) to 80.5 (IQR: 75.75-84.25) (P<0.001) and 57 (53.75-61.25) to 74 (IQR: 70.75- 78.25)(P<0.001), respectively. The states with the highest median RSV for the upper body group were found to be Minnesota (100), Oregon (90), and California (90). The states with the lowest median RSV were found to be Mississippi (47), Nebraska (47), and Louisiana (47) (Figure 4).

**Figure 5:**
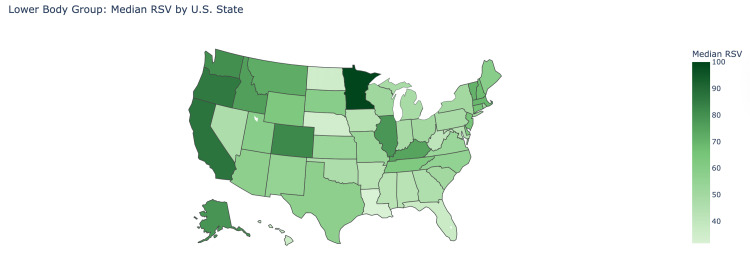
Lower body group: median relative search volume by U.S. state This is a heat map visualization of the United States where median relative search volume (RSV) of the lower body group is shown by how dark the shade of the respective state is according to the legend. The darker the shade of green the higher the median RSV value is in the state.

The median RSV for the overall lower body chronic pain groups in the first interval was 60 (IQR: 0-73), which then rose significantly to 78 (IQR: 50.75-86) in the second interval (P=0.003). Similarly, the chronic knee pain and chronic pelvic pain group’s median RSV rose significantly from 62 (IQR: 58-64) to 77 (70-85.25) (P<0.001) and 73.5 (IQR:72-76.25) to 85.5 (IQR: 83.75-89.25) (P<0.001), respectively (Figure 3). However, the chronic shin pain group showed no statistical difference, where the median RSV stayed at 0 in both intervals (P=0.055). The states that showed the highest median RSV for the lower body group were Minnesota (100), California (87.3), and Colorado (85.7). On the other hand, the states with the lowest median RSV were North Dakota (35), Nebraska (34), and Louisiana (31.7) (Figure 5).

**Figure 6:**
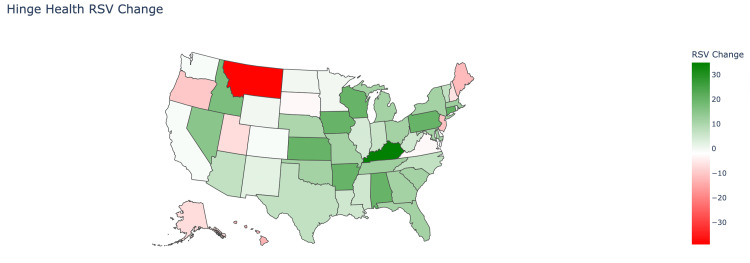
Hinge Health relative search volume change A heatmap of the United States that shows the change in difference of the respective state’s relative search volume (RSV) of “Hinge Health” between the time intervals of January 2020 to August 2022 and September 2022 to April 2025. The darker the shade of green, the greater the increase from one time period to the next. The darker the shade of red, the greater the decrease in RSV value in the state.

Most individual states showed little to no growth and decline in the RSV of Hinge Health between the two intervals. The three states that showed the most significant growth between the two intervals were Kentucky (RSV of 45 to 80), Connecticut (RSV 45 to 66), and Arkansas (18 to 38) (Figure 6). The three states that showed the greatest decrease were Montana (RSV 100 to 61), the District of Columbia (RSV 81 to 61), and Hawaii (RSV 36 to 23).

## Discussion

Our Google Trends analysis revealed a substantial rise in public interest for Hinge Health from 2020 to 2025, with the median relative search volume increasing from 23.5 to 54.5 (P<0.001). States such as Minnesota, California, and Oregon had the most searches. Our study’s results again suggest growing public interest in telehealth platforms for MSK-related pain [14-15]. Our analysis also reflects a transition between the first and second intervals, as there is a significant rise in RSV for Hinge Health, coinciding with the timeline of COVID-19. This pattern parallels patients’ growing familiarity with virtual care during the pandemic, which may help contextualize the persistence of interest after restrictions were lifted. These findings are consistent with previous literature documenting the adoption of telehealthcare for orthopedics, indicating that growing public interest in Hinge Health corresponds with a broader, sustained shift toward digital MSK care models [16]. 

Our study portrayed that states with the highest RSV for Hinge Health, like Minnesota, California, Oregon, and Colorado, are generally urbanized with strong internet access and a dense presence of technological hubs in the vicinity, while ranking high in education and healthcare infrastructure [17]. These characteristics may position such communities for greater engagement with telehealth platforms. In contrast, more rural states with lower RSV, such as Mississippi, Louisiana, Nebraska, and West Virginia, face significant limitations, including internet access, which affects over 20% of rural households, as well as some of the highest rates of illiteracy in the US [18-19]. Such challenges may limit awareness and stymie further adoption of telehealth services in these states, which corresponds with the consistently low RSV in these regions.

Higher RSV states may be more conducive to the adoption of this digital model due to their strong infrastructure and the density of their urban populations [20]. In contrast, lower RSV states may face structural barriers such as poor internet access or a general lack of exposure to these services [21]. These infrastructural gaps may slow the adoption of telehealth despite clinical needs, which raises concerns about the digital divide in healthcare delivery. The states with the highest RSV, Minnesota, California, and Oregon, also rank among the top in household income, education levels, and access to healthcare. In contrast, the findings of this study suggest that lower RSV states may be influenced by ongoing disparities, reinforcing the need for targeted outreach and policy efforts to better support underserved areas [22].

Among states that saw growth in RSV, the urban and rural divide persists. Large rural states like Kentucky and Arkansas experienced a sharp increase in RSV, possibly due to targeted outreach. However, in rural heavy states like Montana, a study identified critical barriers in multiple hospitals, such as workflow redesign, infrastructure upgrades, and a lack of technical support, suggesting that telehealth may be unsustainable. This is consistent with our observation of a drop in interest, which we speculate may be due to inconsistent infrastructure or reduced engagement post-COVID-19 [23,24]. These findings suggest that telehealth is not just about access but also about the population's ability to effectively utilize this service. Without investment in both infrastructure and adult education, platforms like Hinge Health may continue to grow in urban markets while struggling to reach the rural communities that could benefit from them the most.

Despite growing insurer interest in digital MSK platforms, shown by partnerships between Hinge Health and major insurance providers such as Cigna and United Healthcare, several challenges remain [25]. The slow adoption of uniform current procedural terminology codes for digital care creates uncertainty around which services qualify for coverage, and limited integration with the patient’s primary care physician raises concerns about fragmented patient care [26]. Without seamless integration between virtual and in-person providers, patients’ continuity of care may be compromised. However, the high cost and burden of MSK issues make it appealing to employers and payers to adopt digital health solutions. Companies such as Hinge Health report savings of $2,387 per participant and a 2.4x return on investment [27]. These financial benefits may correlate with the observed increase in public interest in digital MSK companies, like Hinge Health.

Looking forward, the question of government-sponsored coverage of digital MSK platforms through Medicare and Medicaid is also critical. While legacy telehealth providers may choose to opt in or out of coverage based on individual evaluations of necessity and economic benefit, permanent federal legislation would be required to integrate coverage of digital health platforms into existing infrastructure [28]. Currently, Medicare permits coverage of non-mental health telehealth services, including digital MSK services, till September 30, 2025 [29]. Although temporary, recent studies show that Medicare beneficiaries using digital MSK platforms report meaningful improvement in pain and function [30]. Therefore, these findings suggest that broader health insurance coverage of clinically validated digital MSK treatments could support greater accessibility and may be associated with the growing interest in digital MSK platforms. 

Our study analyzed temporal and geographical trends in public interest in the online MSK service Hinge Health, but several limitations remain. Google Trends captures search activity rather than actual service use or outcomes, reporting relative interest on a 0 to 100 scale that may be influenced by media or marketing. The absence of demographic and user-level data limits representativeness, as searches may originate from providers, patients, or non-users [12]. The chosen time frame may under- or overestimate true interest; longer study periods could provide interpretable data for excluded regions such as chronic hip, foot, or ankle pain. Missing data were excluded per study criteria. Additional services offered by Hinge Health, including cognitive behavioral therapy, may contribute to search activity but were not evaluated. Internet access disparities further restrict generalizability. Relative search interest also reflects correlation rather than causation, and the lack of longitudinal follow-up prevents linking trends to sustained behavior or service use [12]. Given the normalized and population-level nature of Google Trends data, effect sizes, confidence intervals, and assumption testing were not applied. Although quotation marks were applied in search queries to minimize variability from competing companies or phrasing differences, residual confounding cannot be fully excluded. While Google Trends may give us an idea of the public perception of digital MSK solutions, for further support, it should be supplemented with other research studies with longer study periods and interviews to capture the complete picture of public interest in online MSK services. Despite these limitations, we believe Google Trends data provides key insights for healthcare systems to respond to changes in healthcare delivery and the eventual integration of digital MSK platforms across the country.

## Conclusions

This study demonstrated a significant increase in public interest in Hinge Health’s telehealth services over time across the United States. However, stagnant or declining interest in certain regions reflects disparities in digital infrastructure, healthcare access, socioeconomic status, and cultural attitudes toward virtual healthcare. This study provides compelling evidence to reinforce the growing role and potential of Hinge Health and online MSK treatment platforms in the post-COVID era across many US states and health concerns, while emphasizing that sustained policy action through reimbursement reform, investment in digital infrastructure in rural regions, and community education is critical to achieving equitable access to MSK telehealth nationwide.

## References

[1] Nguyen A, Lee P, Rodriguez EK, Chahal K, Freedman BR, Nazarian A (2025). Addressing the growing burden of musculoskeletal diseases in the ageing US population: challenges and innovations. Lancet Healthy Longev.

[2] Deslauriers S, Déry J, Proulx K, Laliberté M, Desmeules F, Feldman DE, Perreault K (2021). Effects of waiting for outpatient physiotherapy services in persons with musculoskeletal disorders: a systematic review. Disabil Rehabil.

[3] Bailey JF, Agarwal V, Zheng P, Smuck M, Fredericson M, Kennedy DJ, Krauss J (2020). Digital Care for Chronic Musculoskeletal Pain: 10,000 Participant Longitudinal Cohort Study. J Med Internet Res.

[4] Lu L, Gold LS, Koenig KM, Lee JH, Wang G (2024). Digital musculoskeletal program is associated with decreased joint replacement rates. Am J Manag Care.

[5] Wang G, Lu L, Gold LS, Bailey JF (2023). Opioid Initiation Within One Year After Starting a Digital Musculoskeletal (MSK) Program: An Observational, Longitudinal Study with Comparison Group. J Pain Res.

[6] Gormley Gormley, B. (2025, May 22 (2025). Wall Street Journal. Hinge Health goes public, delivering much-needed win for digital-health startups. https://www.wsj.com/articles/hinge-health-goes-public-delivering-much-needed-win-for-digital-health-startups-da641ebc.

[7] Zhang ZY, Tian L, He K (2022). Digital Rehabilitation Programs Improve Therapeutic Exercise Adherence for Patients With Musculoskeletal Conditions: A Systematic Review With Meta-Analysis. J Orthop Sports Phys Ther.

[8] Molina-Garcia P, Mora-Traverso M, Prieto-Moreno R, Díaz-Vásquez A, Antony B, Ariza-Vega P (2024). Effectiveness and cost-effectiveness of telerehabilitation for musculoskeletal disorders: A systematic review and meta-analysis. Ann Phys Rehabil Med.

[9] (2025). McKinsey and Company. Telehealth: A quarter-trillion-dollar post-COVID-19 reality?. https://www.mckinsey.com/industries/healthcare/our-insights/telehealth-a-quarter-trillion-dollar-post-covid-19-reality.

[10] Cohen SA, Cohen LE, Tijerina JD, Bouz G, Lefebvre R, Stevanovic M, Heckmann ND (2021). Google trends as a tool for evaluating public interest in total knee arthroplasty and total hip arthroplasty. J Clin Transl Res.

[11] Mohty KM, Lashkari N, Gittings DJ, Bell JA, Stevanovic M, Nicholson LT (2021). Utilizing Google Trends to track online interest in elective hand surgery during the COVID-19 pandemic. Cureus.

[12] Mavragani A, Ochoa G (2019). Google Trends in infodemiology and infoveillance: methodology framework. JMIR Public Health Surveill.

[13] (2025). Pain Management: Virtual Physical Therapy & More. https://www.hingehealth.com/acquisition/pain-management/.

[14] Bargeri S, Castellini G, Vitale JA, Guida S, Banfi G, Gianola S, Pennestrì F (2024). Effectiveness of Telemedicine for Musculoskeletal Disorders: Umbrella Review. J Med Internet Res.

[15] Haleem A, Javaid M, Singh RP, Suman R (2021). Telemedicine for healthcare: Capabilities, features, barriers, and applications. Sens Int.

[16] Ferorelli D, Moretti L, Benevento M (2022). Digital health care, telemedicine, and medicolegal issues in orthopedics: a review. Int J Environ Res Public Health.

[17] Graves JM, Abshire DA, Amiri S, Mackelprang JL (2021). Disparities in technology and broadband Internet access across rurality: implications for health and education. Fam Community Health.

[18] Anderson Anderson, M. (2018, September 10 (2025). Pew Research Center. About a quarter of rural Americans say access to high-speed internet is a major problem. https://www.pewresearch.org/short-reads/2018/09/10/about-a-quarter-of-rural-americans-say-access-to-high-speed-internet-is-a-major-problem/#:~:text=Fast%2C%20reliable%20internet%20service%20has,survey%20conducted%20earlier%20this%20year..

[19] John Carey, T. (2018, April 14 (2025). Mississippi Encyclopedia. Literacy and Illiteracy. http://mississippiencyclopedia.org/entries/literacty-and-illiteracy/.

[20] Bell N, Hung P, Lòpez-De Fede A, Adams SA (2023). Broadband access within medically underserved areas and its implication for telehealth utilization. J Rural Health.

[21] Cortelyou-Ward K, Atkins DN, Noblin A, Rotarius T, White P, Carey C (2020). Navigating the digital divide: Barriers to telehealth in rural areas. J Health Care Poor Underserved.

[22] Montez JK, Cheng KJ (2022). Educational disparities in adult health across U.S. states: Larger disparities reflect economic factors. Front Public Health.

[23] Karim SA, Tilford JM, Bogulski CA, Hayes CJ, Eswaran H (2025). Exploring telehealth adoption and financial outcomes for rural hospitals during the COVID-19 public health emergency. J Rural Health.

[24] Haque SN, DeStefano S, Banger A, Rutledge R, Romaire M (2021). Factors influencing telehealth implementation and use in frontier critical access hospitals: qualitative study. JMIR Form Res.

[25] (2025). Hinge Health Announces New Collaboration with National Health Plan. https://www.hingehealth.com/resources/press-releases/hinge-health-announces-new-collaboration-with-national-health-plan/.

[26] American Medical Association & Manatt Health. (2023 (2025). Future of Health Commercial Payer Coverage for Digital Medicine Codes. https://www.ama-assn.org/system/files/issue-brief-commercial-payer-coverage-digital-care.pdf.

[27] Hinge Health (2025). Digital musculoskeletal impact on medical claims: 126 employer study. https://www.hingehealth.com/for-organizations/136-employer-study.

[28] (2020). AMCP Partnership Forum: Digital therapeutics-what are they and where do they fit in pharmacy and medical benefits?. J Manag Care Spec Pharm.

[29] (2025). U.S. Department of Health & Human Services. Telehealth policy updates. https://telehealth.hhs.gov/providers/telehealth-policy/telehealth-policy-updates.

[30] O'Connor MI, Dorak Ribaudo M, Peters KC (2025). Clinical efficacy of telemedicine for musculoskeletal conditions in a Medicare Advantage population. Telemed Rep.

